# T. M. McAneny

**Published:** 1972-04

**Authors:** 


### Obituary

### **T. M. McAneny,** BSc, MB, BS, MRCS, LRCP, DA, FFARCS.

Dr. Terence McAneny, Consultant Anaesthetist at Southmead Hospital, Bristol, died suddenly on May 31st, 1971 at the age of 42.

Terence Michael McAneny was a man of exceptional ability who achieved his medical education with the aid of a State Scholarship and an entrance scholarship to the Westminster Hospital. He took a B.Sc. in Physiology in 1950, and combined his first year of clinical studies with a part-time demonstratorship in Pharmacology at St. Bartholomew's. Soon after qualifying in 1953 he began to specialize in anaesthesia, continuing this training during two years in the R.A.M.C., and subsequently holding Registrar and Senior Registrar appointments at the Westminster, Hillingdon and Kingston hospitals. In 1963, at the time of the introduction of the Lucy Baldwin apparatus, he published a valuable and well-documented study of Nitrous Oxide/Oxygen analgesia in obstetrics.

Coming to Bristol in 1964, he at once established himself as a skilful and highly-trained anaesthetist of extensive knowledge and strong intelligence. These qualities, coupled with his cheerful friendliness and energy, soon won him the lasting respect and affection of staff at all levels. In addition, he was a shrewd and thoughtful teacher, who gave great help to a succession of juniors.

To his friends and colleagues, there is a rare and special poignancy in the early death of this very able man, and in the tragic bereavement of his young family. Dr. McAneny was, above all, a devoted husband and father, and we extend our very deepest sympathy to his widow, Margaret, and their four young children.

